# Adjunctive dexamethasone implant in patients with atopic dermatitis and retinal detachment undergoing vitrectomy and silicone oil tamponade: an interventional case series

**DOI:** 10.1186/s12886-019-1094-1

**Published:** 2019-04-03

**Authors:** Ah Ran Cho, Young Hee Yoon

**Affiliations:** 1 Seoul Shinsegae Eye Center, 117, Simin-ro, Uijeongbu-si, Gyeonggi-do Korea; 2 Department of Ophthalmology, University of Ulsan, College of Medicine, Asan Medical Center, 88, Olympic-ro 43-Gil, Songpa-gu, Seoul, 05505 Korea

**Keywords:** Dexamethasone implant, Ozurdex, Retinal detachment, Proliferative vitreoretinopathy, Atopic dermatitis, Silicone oil tamponade

## Abstract

**Background:**

To report the clinical course and outcomes of adjunctive dexamethasone implants in patients with atopic dermatitis (AD) and retinal detachment (RD) undergoing vitrectomy and silicone oil tamponade.

**Methods:**

This retrospective, interventional case series included AD patients with RD and various degrees of proliferative vitreoretinopathy (PVR) who were scheduled to undergo vitrectomy. Following total vitrectomy and retinopexy, silicone oil tamponade was performed. Finally, an intraocular dexamethasone implant was injected intravitreally. Anatomical and functional outcomes were assessed at 12 months, and extended follow-up data were also collected.

**Results:**

Seven eyes from six patients (five male, one female) were included. The median age was 29 (range, 20–38) years. Preoperatively, six eyes were pseudophakic, two eyes had a history of previous vitreoretinal surgery, and one had uveitis. Postoperatively, best-corrected visual acuity improved in two eyes, worsened in one, and remained similar in four. Retinal attachment was maintained in all eyes at 12 months. The major complication was an increase in postoperative intraocular pressure in six eyes, requiring either medical or surgical treatment. During the extended follow-up period (15–37 months), retinas remained attached in all eyes and stable visual acuity was maintained in five.

**Conclusions:**

Injection of an intraoperative dexamethasone implant to silicone oil-filled eyes appears tolerable and may be beneficial in the surgical management of AD patients with RD and PVR.

## Background

Patients with atopic dermatitis (AD) have a high incidence of retinal detachment (RD), which may arise as a result of mechanical trauma and inflammation [1–4]. This specific type of rhegmatogenous RD presents particularly challenging for vitreoretinal surgeons, because it affects younger patients and is associated with higher re-detachment rates and poor prognosis [5, 6]. Studies showed that the major cause of failure following surgery for RD in atopic patients is proliferative vitreoretinopathy (PVR) [6, 7]. Some studies suggest that intraoperative adjuvant steroid injection may reduce the severity of PVR post-surgery [8, 9]. Since chronic intraocular inflammation associated with AD has been proposed as a potential cause of RD and PVR, intraoperative corticosteroid may have an inhibitory effect on PVR in atopic patients [3, 4, 10].

Dexamethasone is a powerful corticosteroid that is five times more potent than triamcinolone and can be used as intravitreal sustained-release dexamethasone implants (DEX implants), a biodegradable posterior segment steroid delivery system [11]. Here, we report our experience with DEX implant injections in conjunction with silicone oil tamponade as an adjuvant in the surgical management of RD in patients with AD.

## Methods

This retrospective, consecutive, observational case series included patients with AD and RD with various degrees of PVR who required RD repair between 2014 and 2016.

All eyes underwent 25-gauge microincision vitrectomy with the Constellation system (Alcon Surgical, Ft. Worth, TX, USA) using a wide-field viewing system. Following a core vitrectomy, complete posterior vitreous detachment was performed. Vitreous traction around the retinal breaks was relieved completely. Vitreous base dissection was completed using scleral indentation. Relaxing retinectomy was performed when necessary. The posterior retina was flattened with perfluorocarbon liquid. Retinopexy was then achieved by laser endophotocoagulation. An air–fluid exchange was performed, and air was exchanged with silicone oil. Finally, a 700 μg DEX implant (Ozurdex®, Allergan, Irvine, CA, USA) was injected intravitreally through the inferior sclera using the 22-gauge applicator device. Cataract surgery was performed in phakic eyes. All surgical procedures were performed by a single surgeon.

Data on demographic characteristics, history of prior ocular surgery, surgical records, and postoperative complications were collected. PVR was graded using the Retina Society Classification System [12]. Best-corrected visual acuity (BCVA), Intraocular pressure (IOP) measured by Goldmann applanation tonometry and anatomical status were assessed at presentation, at 12 months, and at the last follow-up visit. The main outcomes measures were anatomical and functional outcome at 12 months, with longer-term data obtained at the last follow-up visit.

This study was approved by the Institutional Review Board at Asan Medical Center. All examinations and investigations adhered to the tenets of the Declaration of Helsinki.

## Results

The study included seven eyes from six patients (five male, one female) as one patient had bilateral disease (Cases 2 and 5). Baseline clinical characteristics are summarized in Table 1. The median age at the time of surgery was 29 (range, 20–38) years. All eyes had various degrees of PVR, and all except one were pseudophakic. Preoperative visual acuity ranged from hand motion only to 20/25. One case (Case 3) had a history of multiple vitreoretinal procedures, and one other (Case 2) had segmental scleral buckling observed preoperatively on the study eye. One eye (Case 4) showed panuveitis and hypotony prior to surgery. Two cases (Cases 1 and 5) were fellow eyes of those that had received prior surgery for recurrent RD and PVR.

**Table 1:** Baseline patient characteristics

Case	Age decade at the time of surgery, years^a^	Gender	Laterality	Ocular history	Macular status	PVR grade	Pre-operative visual acuity
1	20–29	Male	Right	s/p CE, recurrent PVR in fellow eye	On	A	20/40
2	30–39	Male	Left	s/p CE, s/p SB	Off	B	20/80
3	20–29	Male	Left	s/p CE, s/p multiple PPV	Off	C-2	CF
4	30–39	Female	Right	s/p CE, posterior uveitis with hypotony	Off	A	CF
5	30–39	Male	Right	s/p CE, recurrent PVR in fellow eye	On	B	20/25
6	20–29	Male	Right	Cataract	On	A	20/400
7	30–39	Male	Right	s/p refractive surgery, s/p CE	Off	C-3	HM

*s/p* status post, *PVR* proliferative vitreoretinopathy, *Gr* grade, *CE* cataract extraction, *SB* scleral buckle, *PPV* pars plana vitrectomy, *CF* counting fingers, *HM* hand motion

^a^Ages were presented as age decade to preserve patients’ anonymity

At 12 months, retinal attachment had been maintained in all eyes, although Case 7 required an additional procedure for a recurrent inferior RD at 1 month. Postoperative BCVA improved in two eyes (Cases 6 and 7), worsened in one eye (Case 2), and remained similar in four eyes (Cases 1, 3, 4, and 5). During the initial 12 month follow-up period, silicone oil was removed in two eyes (Cases 5 and 6), resulting in maximal improvement in visual acuity. Although the retinas were seen to be well attached in the other eyes, we opted not to remove silicone oil unless emulsification or a significant increase in intraocular pressure (IIOP) arose. We intended to leave the silicone oil in place for as long as possible in case the patient had lost vision in the fellow eye despite multiple surgeries for recurrent RD with PVR (Cases 1 and 5). In all cases, retinas also remained attached during the extended follow-up period (15–37 months). Visual acuity (VA) was maintained until the final visit in all but two cases (Cases 1 and 4), which showed a mild decrease in VA due to recurrent uveitis and IIOP.

**Fig. 1:**
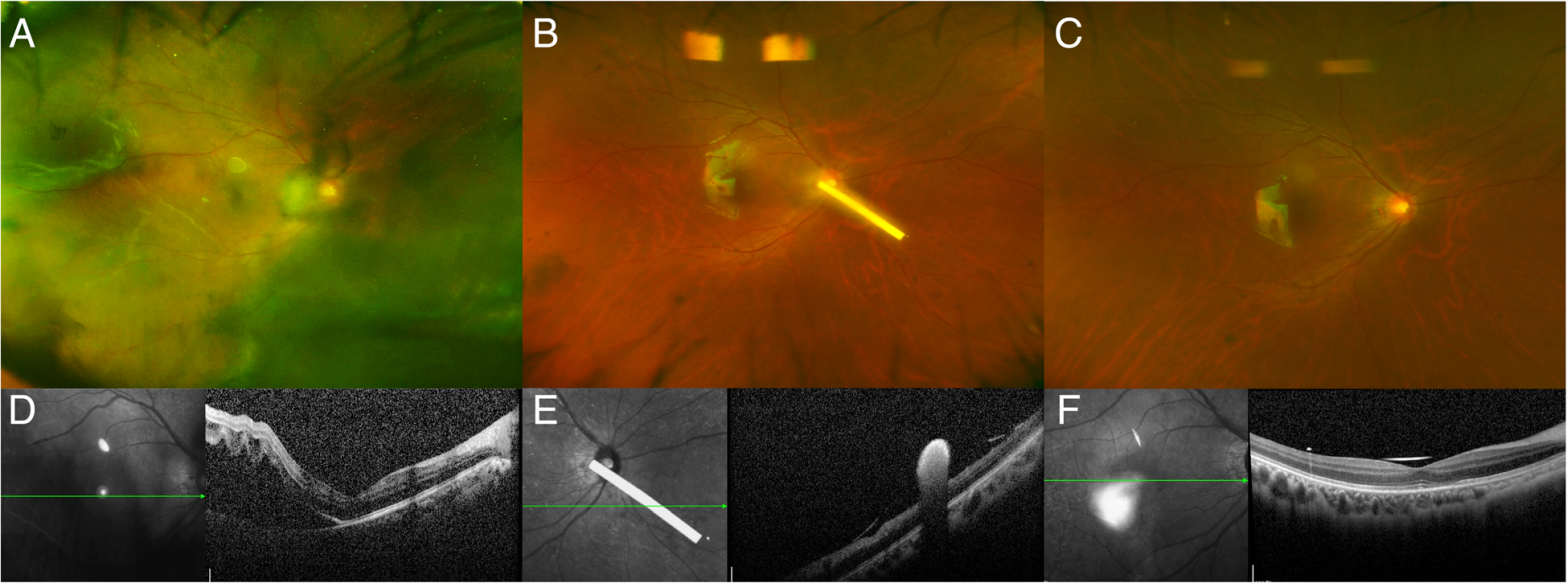
Pre- and postoperative images of Case 1. **a** Preoperative wide-angle fundus photograph showing inferotemporal retinal detachment with vitreous opacity. **b** Fundus at 3 days post-surgery showing attached retina and DEX implant located near the optic disc. **c** Fundus at 4 weeks post-surgery showing successfully attached retina with silicone oil tamponade. Note that the implant has spontaneously moved to the periphery. **d** Preoperative optical coherence tomography showing retinal detachment involving the fovea. **e** Optical coherence tomography obtained at 3 days post-surgery. **f** Optical coherence tomography obtained at 4 weeks postsurgery

**Fig. 2:**
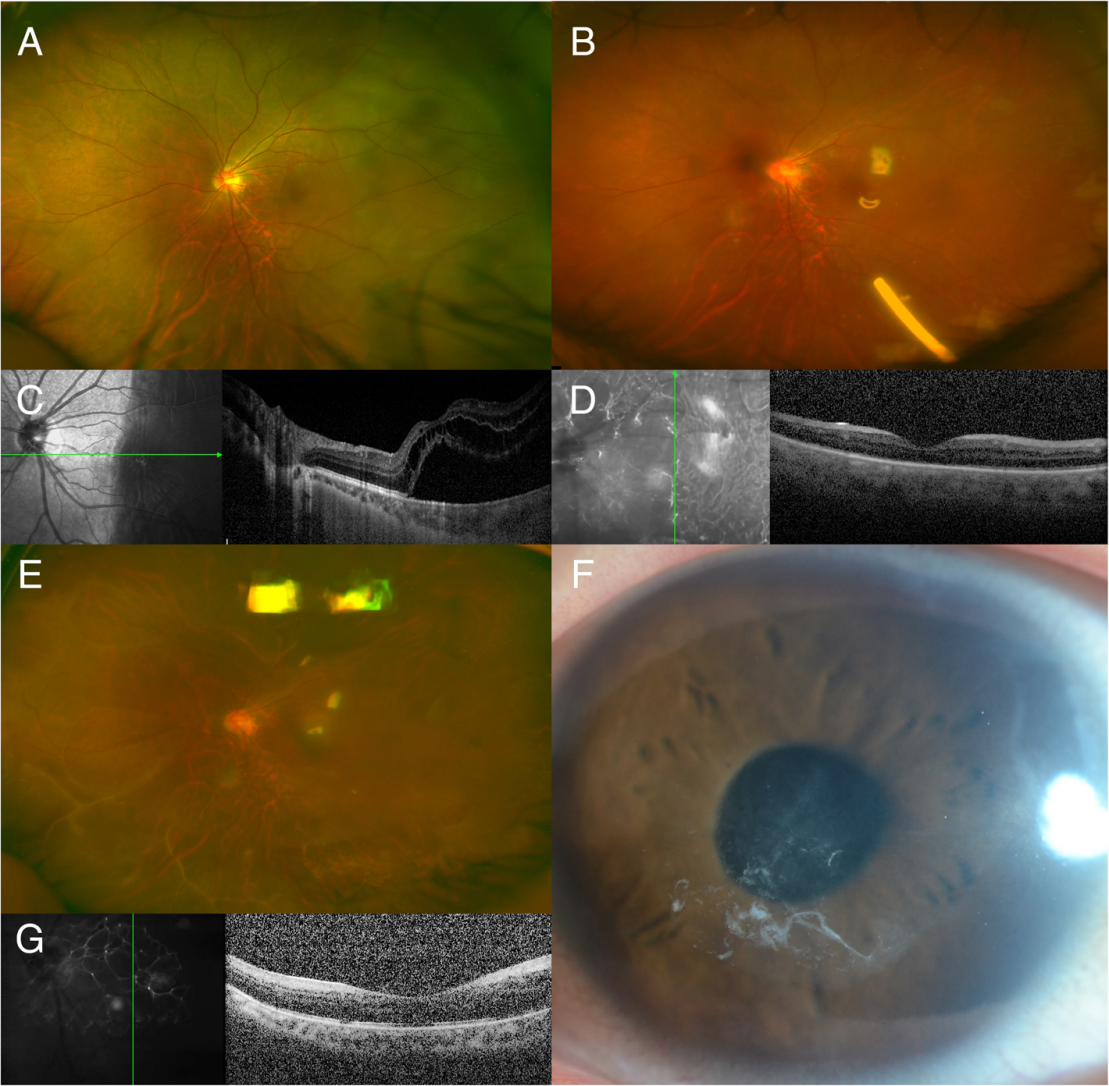
Pre- and postoperative images of Case 2, who had undergone prior scleral buckle surgery. **a** Preoperative wide-angle fundus photograph showing recurrent retinal detachment despite previous segmental scleral buckling surgery. **b** Fundus at 2 weeks post-surgery showing an attached retina and a dexamethasone implant in silicone oil. The photo is blurry because of a corneal epithelial defect. **c** Preoperative optical coherence tomography image showing retinal detachment involving the fovea. **d** Optical coherence tomography image obtained at 2 weeks post-surgery. **e** Wide-angle fundus photograph obtained at 1 year post-surgery. The image is hazy because of herpetic keratitis. **f** Slit-lamp photograph showing central corneal opacity. **g** Optical coherence tomography image obtained at 1 year post-surgery. Although the retina was successfully attached, visual acuity failed to improve because of corneal opacity

**Fig. 3:**
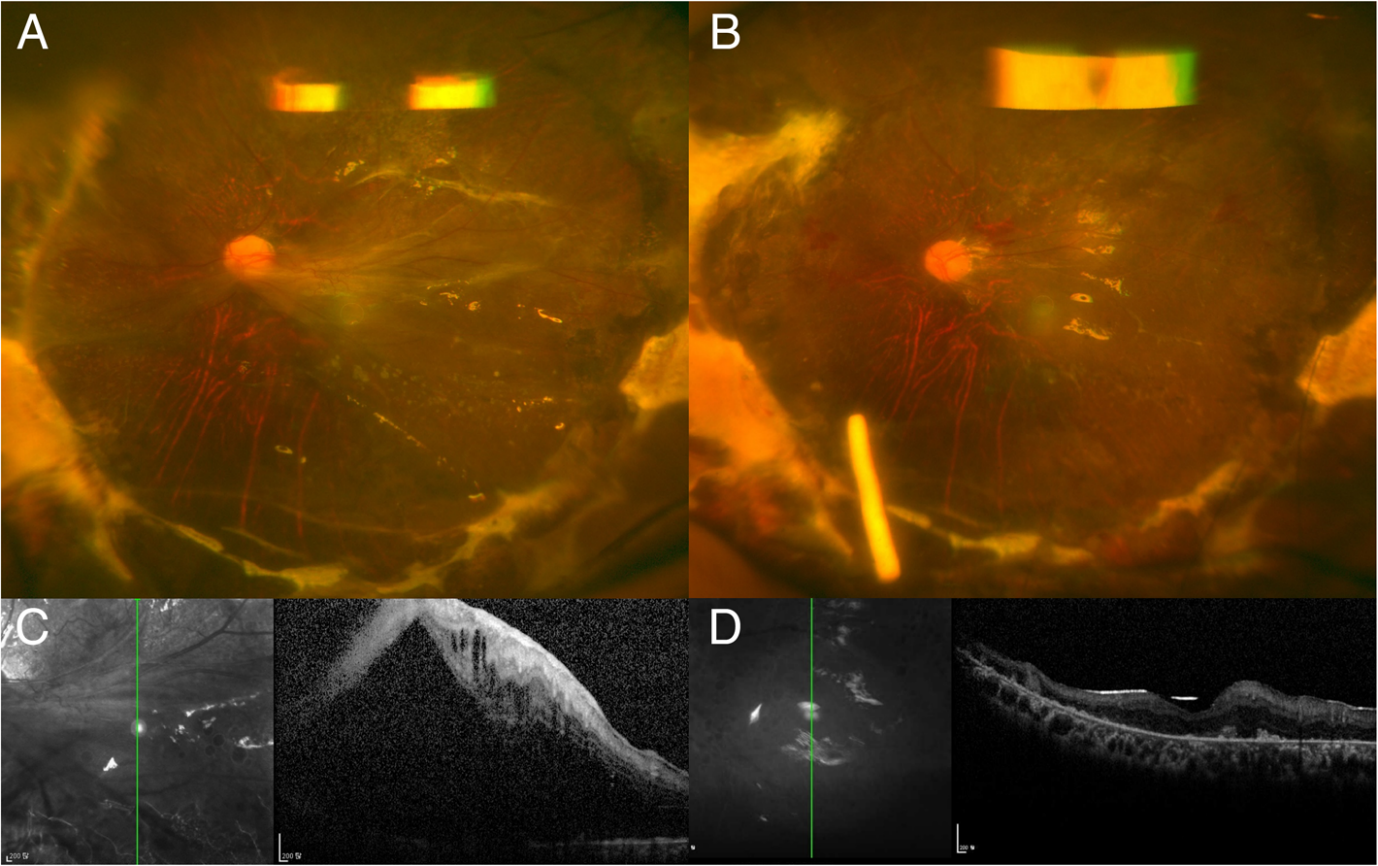
Pre- and postoperative images of Case 3 who had undergone multiple prior vitrectomy procedures. **a** Preoperative wide-angle fundus photograph showing proliferative vitreoretinopathy with silicone oil tamponade. **b** Postoperative wide-angle fundus photograph showing successful attachment of the retina and DEX implant located in the silicone oil. **c** Preoperative optical coherence tomography showing retinal thickening and retinal detachment involving the fovea. **d** Postoperative optical coherence tomography showing the reattached retina

Figure 1 shows the wide-angle fundus photo and spectral domain optical coherence tomography before and after surgery in Case 1. This patient presented with rapidly progressing RD and early PVR within days of symptom development. On the day after surgery, the DEX implant was located near the optic disc, but it moved spontaneously to the inferior periphery within a few days. Figure 2 shows successful retinal reattachment in a patient after failed scleral buckle surgery. Unfortunately, his vision failed to improve because of herpetic keratitis. Figure 3 shows good anatomic attachment of the retina without recurrent PVR in a patient (Case 3) who had a history of multiple vitrectomies for recurrent RD with grade C-2 PVR. Despite good anatomic results, his vision failed to notably improve.

Postoperative IOP elevation (> 21 mmHg) occurred in six eyes. One eye (Case 3) showed pupillary block at postoperative day 1, which completely resolved after surgical peripheral iridotomy (7 mmHg without medication at the last follow-up). One eye (Case 5), which exhibited preoperative IIOP (22 mmHg with two IOP-lowering medications), required Ahmed valve implantation surgery 1 month post-vitrectomy (14 mmHg one medication at the last follow-up). Four eyes without preoperative IOP increase (Cases 1, 2, 4, and 7) developed glaucoma, which was confirmed by a glaucoma subspecialist. While three eyes (Cases 1, 2 and 7) were successfully managed medically (≤ 21 mmHg with one or two medications), one eye (Case 4) required Ahmed valve implantation (18 mmHg with three medications at the last follow-up).

Silicone oil was removed in two eyes but left in the other five eyes until the last follow-up, primarily because the patients were reluctant to undergo oil removal because of fear of re-detachment. Among the five eyes in which silicone oil was maintained, one case (Case 4, who presented with preoperative panuveitis) showed recurrent cystoid macular edema (CME) after degradation of the DEX implant. Repeat injection of a DEX implant was performed into the silicone oil-filled vitreous, which resolved the CME. Silicone oil emulsification or increase of intraocular pressure due to persistent silicone oil tamponade was not noted. Postoperative endophthalmitis was not observed in any cases. The outcomes and complications of all cases are summarized in Table 2.

**Table 2:** Surgical treatments and outcomes at 12 months and final follow-up

Case	Visual acuity at 12 months	Retinal status at 12 months	Silicone oil status at 12 months	Postoperative complications during the first 12 months	Final follow-up duration, months	Final visual acuity	Final retinal status
1	20/50	Attached	In	Recurrent uveitis IIOP – topical treatment	33	20/100	Attached
2	20/400	Attached	In	Herpetic keratitis IIOP – topical treatment	30	20/400	Attached
3	HM	Attached	In	IIOP – surgical iridotomy	37	HM	Attached
4	CF	Attached	In	Recurrent CME IIOP – Valve implantation	32	CF	Attached
5	20/25	Attached	Out	IIOP – Valve implantation	23	20/25	Attached
6	20/40	Attached	Out	None	18	20/30	Attached
7	20/100	Attached	In	Inferior RD – PPV IIOP – topical treatment	15	20/100	Attached

*IIOP* increased intraocular pressure, *HM* hand motion, *CF* counting fingers, *CME* cystoid macular edema, *RD* retinal detachment, *PPV* pars plana vitrectomy

## Discussion

In this study, we evaluated the long-term outcome of adjunctive DEX implants in patients with AD and PVR complicated by RD, who had undergone vitrectomy and silicone oil tamponade. At 12 months post-surgery, anatomical and functional outcomes were favorable in all patients, and this was maintained throughout the extended follow-up period.

Several hypotheses for the pathogenesis of RD associated with AD have been suggested, including trauma (e.g., repeated slapping or rubbing of the eye), which may result in breaks in the anterior retina [1]. Takahashi et al. suggested that retinal breaks could be related to the intense inflammation produced by the abnormal immune reaction associated with atopy [10]. Although not proven, some investigators suggested that AD involves a dysfunctional immune response to certain neuroectodermal-derived antigens, such as those of the skin, lens, vitreous, and retina [4, 13].

Despite the outcomes of RD surgery have improved significantly over the recent years, multiple procedures are frequently required to achieve the final reattachment in patients with AD [5]. There are many factors that contribute to the recurrence of RD in atopic patients, including the presence or development of PVR and new retinal breaks [6]. Fong et al. suggested that high re-detachment rates in atopic patients could be due to persistent chronic inflammation leading to PVR, or inflammation of the ciliary body causing traction to the peripheral retina, which can result in new breaks [5].

As corticosteroids reduce intraocular inflammation and suppress cellular proliferation [14, 15], they may be a useful additional tool in the treatment of PVR. A randomized trial demonstrated that systemic corticosteroids can reduce the early stages of PVR after RD surgery [16]. However, the results of previous studies evaluating the role of triamcinolone acetonide as adjunctive medication for PVR are inconsistent [9, 17]. Cheema et al. suggested that triamcinolone acetonide may have potential for RD with PVR [9]. However, a randomized trial showed that the outcome of established PVR was not significantly improved by adjunctive triamcinolone acetonide injection in silicone-filled eyes [17].

Dexamethasone is a corticosteroid that is five times more potent than triamcinolone [11]. The slow-release DEX implant was shown to maintain high dexamethasone concentrations during the first 2 months post-injection, with lower concentrations sustained for up to 6 months [18]. Although the efficacy of this agent has been well determined for various retinal diseases, previous studies demonstrated no significant difference in anatomic retinal reattachment and PVR recurrence rates after implant injection [8, 19]. In addition, a recent randomized, controlled trial showed that primary anatomic success for PVR was not improved significantly by DEX implants in silicone-filled eyes [19]. However, the proportion of patients requiring more than one surgical procedure or the proportion of eyes with macular edema was significantly reduced in the adjunctive DEX implant group [19].

In the current study, we explored the hypothesis that, in patients with AD, dexamethasone may suppress the persistent postoperative inflammatory reaction in PVR-associated RD. As anticipated, postoperative inflammation was mild in most cases, contributing to the low rate of recurrent PVR. However, in one pseudophakic eye that exhibited grade C-3 PVR with total preoperative RD, inferior RD developed during the early postoperative period. Because inflammation was not severe in this DEX implant-treated eye, the retina was reattached with only minor revision. Hida et al. demonstrated that PVR is more common in eyes with previous cataract surgery associated with AD [6]. Moreover, PVR at the time of initial presentation in atopic patients was seen to make reattachment more challenging [6].

Major concerns associated with the intraocular injection of corticosteroids are the risk of cataract formation and increased IOP. Most eyes in the present case series were pseudophakic, and one phakic eye underwent cataract extraction as part of the surgical management of RD. Therefore, the cataractogenic effect of the DEX implant in silicone-filled eyes need not be a concern. Elevated IOP has been reported in 39.2% of eyes undergoing adjunctive DEX implant injection in silicone-filled eyes, which is a higher rate than that seen in non-adjunctive silicone oil-filled eyes [19]. However, in that study there was no statistically significant difference between the adjunct and control groups regarding the development of glaucoma [19]. The proportion of patients with increased IOP and glaucoma was greater in the present series, probably due to the additional risk factor of AD [20]. While the immunosuppressive effect of steroids poses the risk of postoperative infectious endophthalmitis, no cases were observed in the present series.

The main limitation of this study is the retrospective design and low patient number due to the low incidence of AD. This case series may be considered as a pilot study, showing the feasibility of intravitreal DEX implants during surgery for RD in patients with AD. The strengths of the study include the duration of the follow-up period and single center/single surgeon design, which reduces the influence of external factors.

## Conclusions

In conclusion, the results of this study are encouraging, showing promise for achieving a favorable success rate for retinal reattachment in patients with AD. However, close postoperative monitoring and management is recommended to avoid potential complications associated with dexamethasone, such as postoperative IOP increase. Although the results of this pilot series require confirmation in future randomized prospective studies, adjunctive DEX implants in conjunction with silicone oil tamponade implants may be a useful additional tool in the treatment of atopic patients with RD associated with PVR.

## Abbreviations

AD: Atopic dermatitis
BCVA: Best-corrected visual acuity
CME: Cystoid macular edema
DEX: Dexamethasone
IIOP: Increased intraocular pressure
IOP: Intraocular pressure
PVR: Proliferative vitreoretinopathy
RD: Retinal detachment
VA: Visual acuity
